# Supplementary material for: Triple Network Resting State Connectivity Predicts Distress Tolerance and Is Associated with Cocaine Use
Source: J Clin Med. 2019 Dec 3;8(12):2135. doi: 10.3390/jcm8122135 (PMC6947426; doi:10.3390/jcm8122135)
Supplement: Supplementary file 1 [file jcm-08-02135-s001.pdf]

Table S1. Cox regression model examining effect of potential covariates on distress tolerance.

	χ^2	*OR*	*95% CI*	
			**Lower**	**Upper**
Step 1	14.75			
Age		1.07	0.99	1.15
IQ		0.98	0.93	1.03
Gender		1.51	0.44	5.22
Race$_{\text{(Black/AA)}}$		1.05	0.25	4.48
Task 1 Motivation		0.86	0.63	1.18
Task 2 Motivation		1.21	0.88	1.67
Task 1 Performance		0.70	0.42	1.18
Task 2 Performance		1.28	0.76	2.17
DT Task Order $_{\text{(MT First)}}$		2.30	0.79	6.68
Anxiety Symptoms		1.08	0.86	1.36
Depressive Symptoms		1.00	0.87	1.14

Note: *OR* = odds ratio; **p* < 0.05, ***p* < 0.01, ****p* < 0.001.

Table S2. Cox regression models for (a) within rsFC, (b) between rsFC, (c) post-hoc L.RAI, and (d) combined within and between rsFC.

S2a)

	χ^2	*OR*	*95% CI*	
			Lower	*Upper*
DMN				
Step 1	2.57			
Group		0.92	0.48	1.77
DMN		1.30	0.92	1.83
Step 2 (Δ from step 1)	0.07			
Group		0.94	0.48	1.86
DMN		1.40	0.74	2.63
DMN × Group		0.91	0.43	1.91
SN				
Step 1	0.57			
Group		0.80	0.42	1.51
SN		1.06	0.76	1.48
Step 2 (Δ from step 1)	0.89			
Group		0.80	0.42	1.53
SN		1.19	0.79	1.79
SN × Group		0.72	0.36	1.42
RECN				
Step 1	2.24			
Group		0.77	0.40	1.47
RECN		1.27	0.90	1.79
Step 2 (Δ from step 1)	1.90			
Group		0.72	0.36	1.43
RECN		1.03	0.67	1.58
RECN × Group		1.62	0.82	3.23
LECN				
Step 1	**8.53***			
Group		0.96	0.50	1.85
LECN		**0.63****	**0.45**	**0.87**
Step 2 (Δ from step 1)	1.18			
Group		1.01	0.51	2.03
LECN		**0.54****	**0.36**	**0.82**
LECN × Group		1.45	0.73	2.90

Note: OR = odds ratio; *p < 0.05, **p < 0.01, ***p < 0.001.

S2b)

	χ^2	*OR*	*95% CI* *Lower*	*Upper*
R.RAI				
Step 1	1.07			
Group		0.81	0.43	1.54
R.RAI		0.88	0.64	1.21
Step 2 (Δ from step 1)	2.13			
Group		0.83	0.42	1.61
R.RAI		0.71	0.45	1.11
R.RAI × Group		1.65	0.83	3.29
L.RAI				
Step 1	**6.36***			
Group		0.67	0.34	1.30
L.RAI		**0.66***	**0.48**	**0.91**
Step 2 (Δ from step 1)	0.11			
Group		0.69	0.35	1.36
L.RAI		0.62	0.37	1.04
L.RAI × Group		1.12	0.58	2.16

Note: *OR* = odds ratio; *$p < 0.05$, **$p < 0.01$, ***$p < 0.001$.

S2c)

	χ^2	*OR*	*95% CI* *Lower*	*Upper*
DMN-SN				
Step 1	**6.53***			
Group		0.73	0.38	1.39
DMN-SN		**1.65****	**1.12**	**2.43**
LECN-SN				
Step 1	3.76			
Group		0.71	0.37	1.37
LECN-SN		0.73	0.52	1.03
LECN-DMN				
Step 1	0.45			
Group		0.81	0.42	1.54
LECN-DMN		1.00	0.73	1.35

Note: *OR* = odds ratio; *$p < 0.05$, ** $p < 0.01$, ***$p < 0.001$.

S2d)

	χ^2	*OR*	*95% CI* *Lower*	*Upper*
Step 1	**12.64****			
Group		0.91	0.47	1.78
LECN		**0.64****	**0.46**	**0.89**
DMN-SN		**1.70***	**1.11**	**2.61**

Note: OR = odds ratio; *p < 0.05, **p < 0.01, ***p < 0.001.

Table S3. Cocaine use severity predicting (a) distress tolerance and (b) rsFC.

S3a)

	χ^2	*OR*	*95% CI* *Lower*	*Upper*
Amount of Cocaine Use Per Using Day				
Step 1	0.26	1.48	0.33	6.72
Cocaine Use Frequency				
Step 1	0.01	0.98	0.58	1.65

Note: OR = odds ratio; *p < 0.05, **p < 0.01, ***p < 0.001.

S3b)

	R^2	*B*	*SE*	*95% CI* Lower	*95% CI* Upper
**Amount of Cocaine Use Per Using Day**					
LECN	0.01	0.26	0.71	−1.19	1.72
**DMN-SN**	**0.19**	**1.15**	**0.47**	**0.18**	**2.12**
**Cocaine Use Frequency**					
LECN	0.08	−1.72	1.15	−4.08	0.65
DMN-SN	0.01	0.34	0.88	-1.47	2.16

Note: $^*p < 0.05$, $^{**}p < 0.01$, $^{***}p < 0.001$.

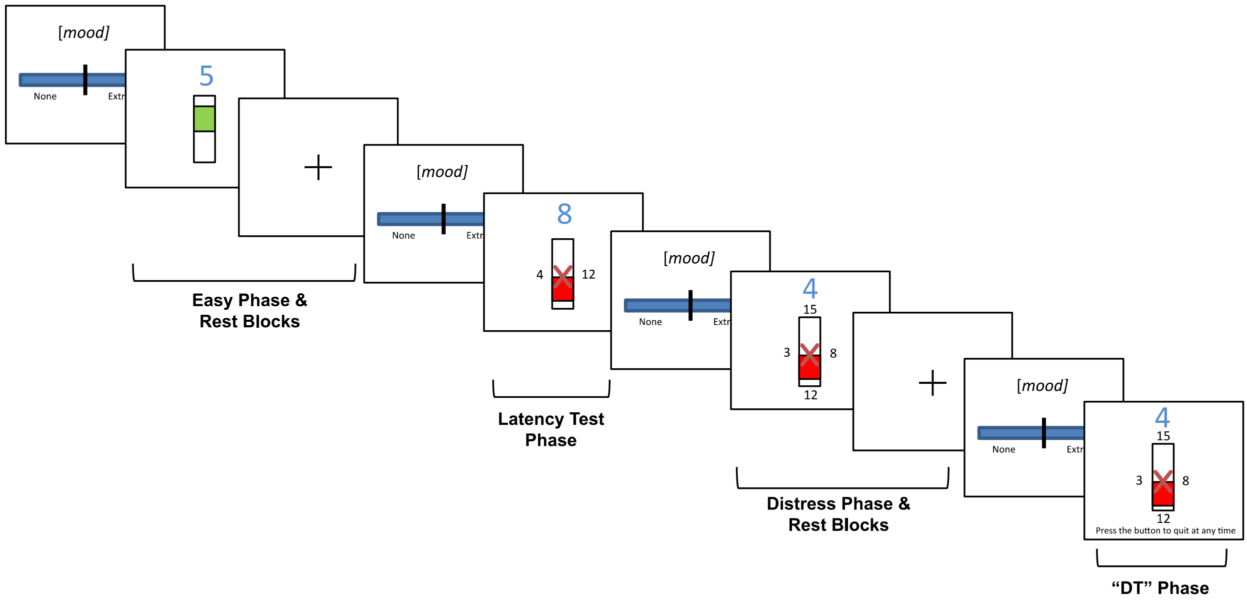

Figure S1a. Task design for the fMRI version of the Paced Auditory Serial Addition Task (PASAT-M). The PASAT-M is a 10-minute block design task consisting of four phases: easy, latency, distress, and distress tolerance. A series of numbers flash on the screen one at a time and the participant is instructed to add the current number on the screen to the previously presented number, using an MRI compatible joystick to indicate the correct answer from four possible choices before the subsequent number appears. Throughout each phase, correct responses result in a pleasant bell sound and a one-point increase in the participants' score. During the latency and hard phases, incorrect and/or slow responses result in an explosion noise and a one-point decrease in points. Task difficulty (i.e., time between number presentations) is titrated to participant skill level during the 5-minute latency phase. During the final two phases, the latency between number presentations is set to 2.5× greater the participant's calculated skill level, resulting in forced failure. During the DT phase, participants are instructed that they can win back points and no longer lose points for incorrect responses, yet are given the option to quit the task at any time until the task terminates on its own after 10 minutes , but are incentivized to persist for the duration of the DT phase as they are told that the monetary compensation they will receive is contingent upon task performance across all phases of the task. Performance on this task is defined as the latency between number presentation as determined during the latency task phase.

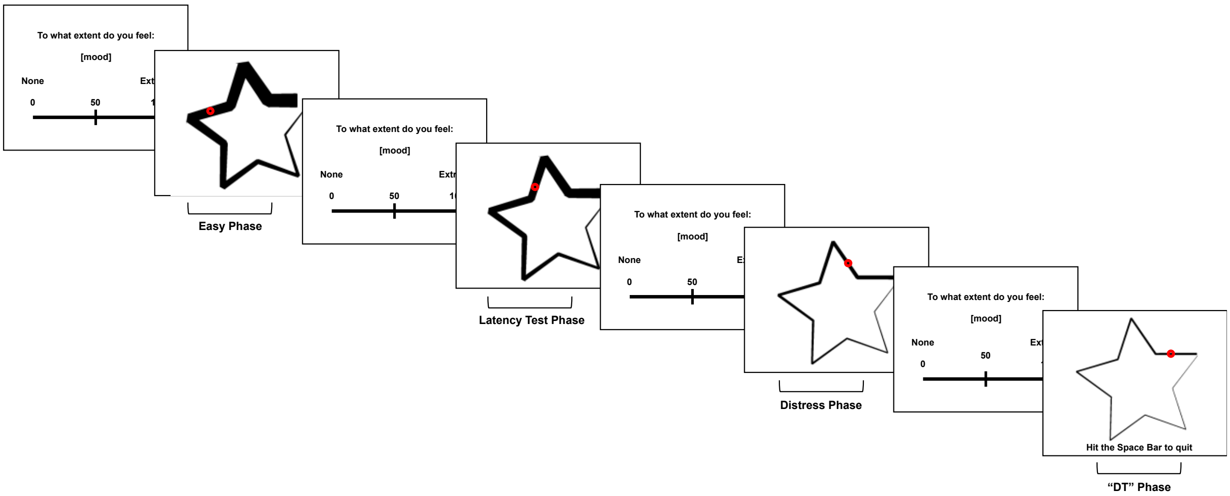

Figure S1b. Task design for the fMRI version of the Mirror Tracing Persistence Task (MTPT-M). adapted from the computerized version of the Mirror Tracing Persistence Task (MTPT-C; Strong et al., 2003). A series of star figures are presented on the computer screen one at a time and participants are instructed to trace a red dot along the tapered edge of the star using an fMRI compatible joystick which is programmed to move the dot in the opposite direction. Errors occur when the red dot is moved too slowly or tracks outside of the star's boundary, both of which result in aversive auditory feedback (i.e., buzzer sound) and require participants to start tracing from the beginning. The task is a block design with four phases (i.e., easy, latency, distress, and distress tolerance), and difficulty is titrated to participant skill level during the latency phase and applied during the final two phases. Difficulty is adjusted by tapering the width of the star edge and adjusting the speed of the red dot. During the final, DT phase, participants are told they will no longer lose points for errors and can only win back points. Participants are given the option to quit the task at any time until the task terminates on its own after ten minutes, and participants are incentivized to persist for the duration of the DT phase as they are told that the monetary compensation they will receive is contingent upon task performance across all phases of the task. Performance on the MTPT-M is defined as the mean cursor speed (i.e., red dot) during the latency phase. .
